# Pediatric morphometric study to guide the optimized implantation of the Osia^®^ 2 implant system

**DOI:** 10.1007/s00405-022-07338-2

**Published:** 2022-03-16

**Authors:** Balint Posta, Adam Perenyi, Linda Szabo, Roland Nagy, Gabor Katona, Zsuzsanna Csakanyi, Laszlo Rovo, Zsofia Bere

**Affiliations:** 1 Department of Oto-Rhino-Laryngology and Head-Neck Surgery, University of Szeged, Szeged, Hungary; 2 Ear Nose Throat Department, Heim Pal National Pediatric Institute, Budapest, Hungary

**Keywords:** Osia, Conductive hearing loss, Mixed-type hearing loss, Pediatric surgery

## Abstract

**Purpose:**

Continuous technological advances result in the availability of new bone conduction hearing implants, of which their suitability for pediatric patients is of major concern. The Cochlear^TM^Osia^®^ 2 is a new active osseointegrated steady-state implant system that uses digital piezoelectric stimulation to treat hearing loss. The implant in the United States was approved for patients aged 12 years and above, whereas the CE mark is independent of age, the only requirement is body weight of at least 7 kg. Therefore, further clinical studies are required to assess device characteristics in younger patients. The aim of our study was to perform a morphometric study among 5–12-year-old children, and to develop a surgical protocol for Osia 2 system implantation based on these findings.

**Methods:**

We examined retrospectively cranial CT scans of 5–12-year-old patients from our clinical database. We measured the bone and soft-tissue thickness in the region of interest, and the position of the sigmoid sinus. 3D printed temporal bones were also used for planning.

**Results:**

Soft-tissue thickness varied between 3.2 ± 0.5 mm and 3.6 ± 0.6 mm and bone thickness varied between 3.5 ± 1.1 mm and 4.7 ± 0.3 mm. The sigmoid sinus was located 1.3 ± 0.2 cm posterior to the ear canal, and the anterior distance was 4.8 ± 0.9 to 7.1 ± 1.1 mm.

**Conclusions:**

Our morphometric studies showed that patients aged 5–12 have different anatomical dimensions compared to adults, but that implantation of the Osia 2 system is feasible in these patients using an altered implant positioning recommended by our data. The Cochlear™ Osia^®^ 2 is, therefore, an option for hearing rehabilitation in younger pediatrics.

## Introduction

Since osseointegration was developed [1, 2] and the first direct bone conduction hearing aid was implanted by Professor Tjellström in 1977, the application of bone conduction implants (BCI) has increased exponentially, and extensive development of the device has begun [3–6]. Considering the needs and advantages of early hearing rehabilitation, pediatric application of BCI is also evolving. However, date of surgery, type of implanted system, applied surgical techniques, and complication management in small children is always a major concern [7]. Therefore, stable, safe, high-power implants and straight-forward surgery adapted for the pediatric population is necessary.

**Fig. 1:**
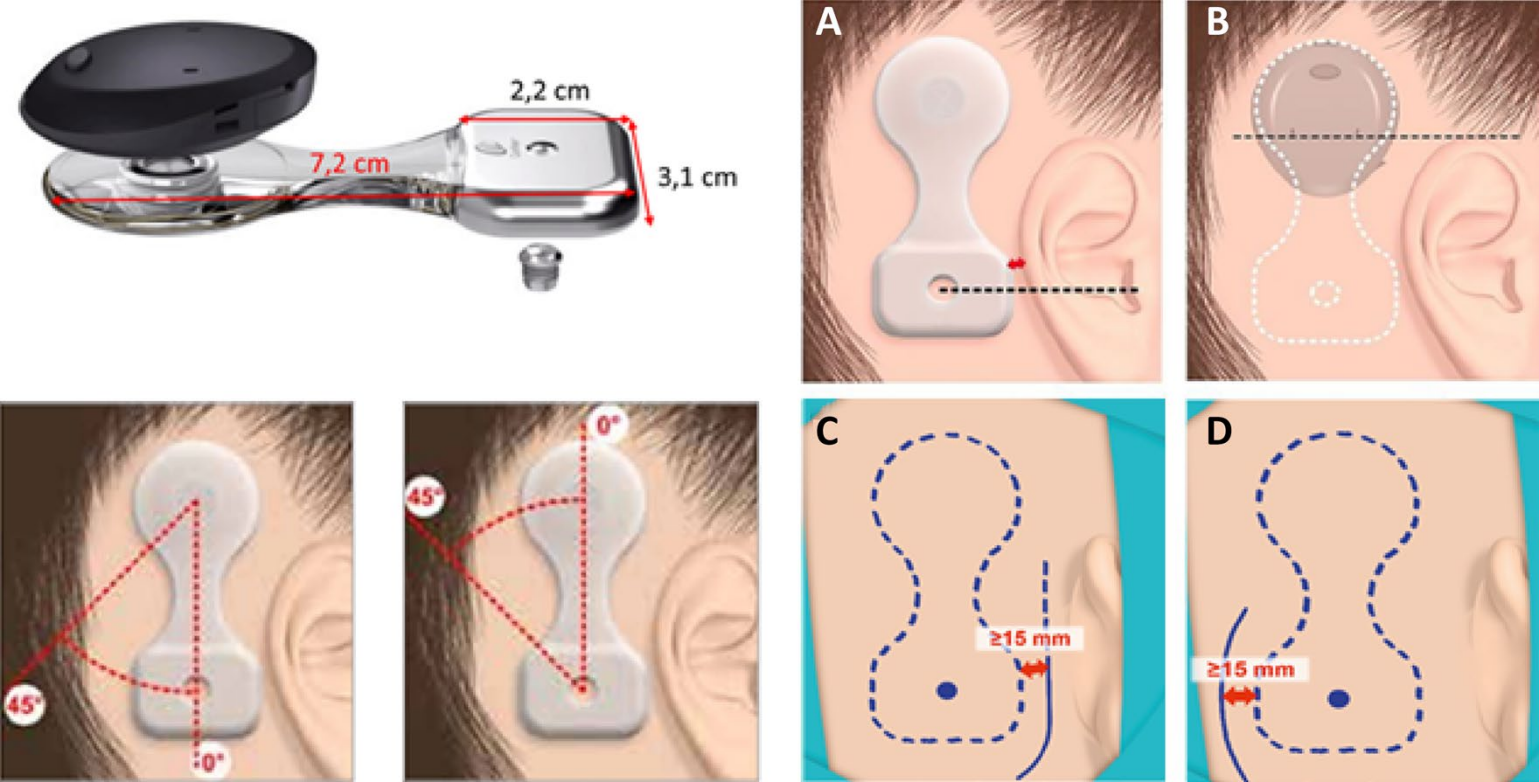
Osia 2 system implant with size parameters (top left) and manufacturer recommended position (bottom left panels and panels **A**–**D**). Copyright © Cochlear Ltd. All rights reserved. Illustrations provided courtesy of and with permission from Cochlear

The Cochlear Osia 2 system is a new active, transcutaneous, osseointegrated steady-state implant system that uses digital piezoelectric stimulation (Fig. 1). The Osia system is intended for adults and children with conductive or mixed hearing loss (up to 55 dB HL) and single-sided sensorineural deafness (SSD). The piezoelectric transducer is fixed to the bone via the BI300 titanium implant, and signal is transferred between implant and sound processor (SP) via a digital RF link [8, 9]. The Osia system grants high-power output and improved high frequency gain for optimizing speech perception and compared to Baha^®^ 5 Power it provides significantly higher functional gain at higher frequencies (5–7 kHz) [10, 11]. Since it is transcutaneous, the possibility of trauma and soft-tissue complications is lower compared to percutaneous BCIs, and it is aesthetically more feasible [12]. Considering the audiological and safety benefits of active transcutaneous systems, early application of these devices in the pediatric population would be advantageous. At present, the implant in the United States is approved for patients aged 12 years and above, whereas the CE mark is independent of age, the only requirement is body weight of at least 7 kg. Since children are smaller and have morphological differences, i.e., thinner soft-tissue and bone structure compared to adults [13, 14], further clinical study of the implant area is required. Intraoperative complications, such as exposing the dura, injury of the sigmoid sinus and consequent bleeding, or entering the mastoid cavity can also be avoided via prudent implantation technique. Therefore, knowledge of age-dependent anatomy is essential. The aim of our study was to perform a morphometric investigation among 5–12-year-old children to develop a safe surgical protocol for implantation of the Osia 2 system.

## Materials and methods

The ethical approval for this study was obtained from the Institutional Review Board (Human Investigation Review Board, University of Szeged, Albert Szent-Györgyi Clinical Centre (Reference number: 164/2020). In this retrospective study high resolution cranial CT scans with a minimum of 0.625 mm slice thickness (0.4 mm in a proportion of cases) of 40 children between the ages of 5 and 12 years were collected from our clinical database and systematically analysed. The indication of cranial CT were the following: polytrauma/whole body CT (with no trauma in the region of interests) (*n* = 15); uni- or bilateral external ear canal atresia without additional severe craniofacial malformation or syndrome (*n* = 6); temporal CT prior to ear surgery (*n* = 8), and preoperative cranial CT for neurosurgery patients or cranial CT for patients with particular neurological disorder (*n* = 11). Scans from individuals with any form of head trauma including temporal region or severe complex craniofacial malformations were excluded. Different attributions of the retroauricular/temporoparietal region were measured in four specified age groups (5–6 years; 7–8 years; 9–10 years, and 11–12 years). The number of patients in each group was 10, and both male and female candidates were selected.

**Fig. 2:**
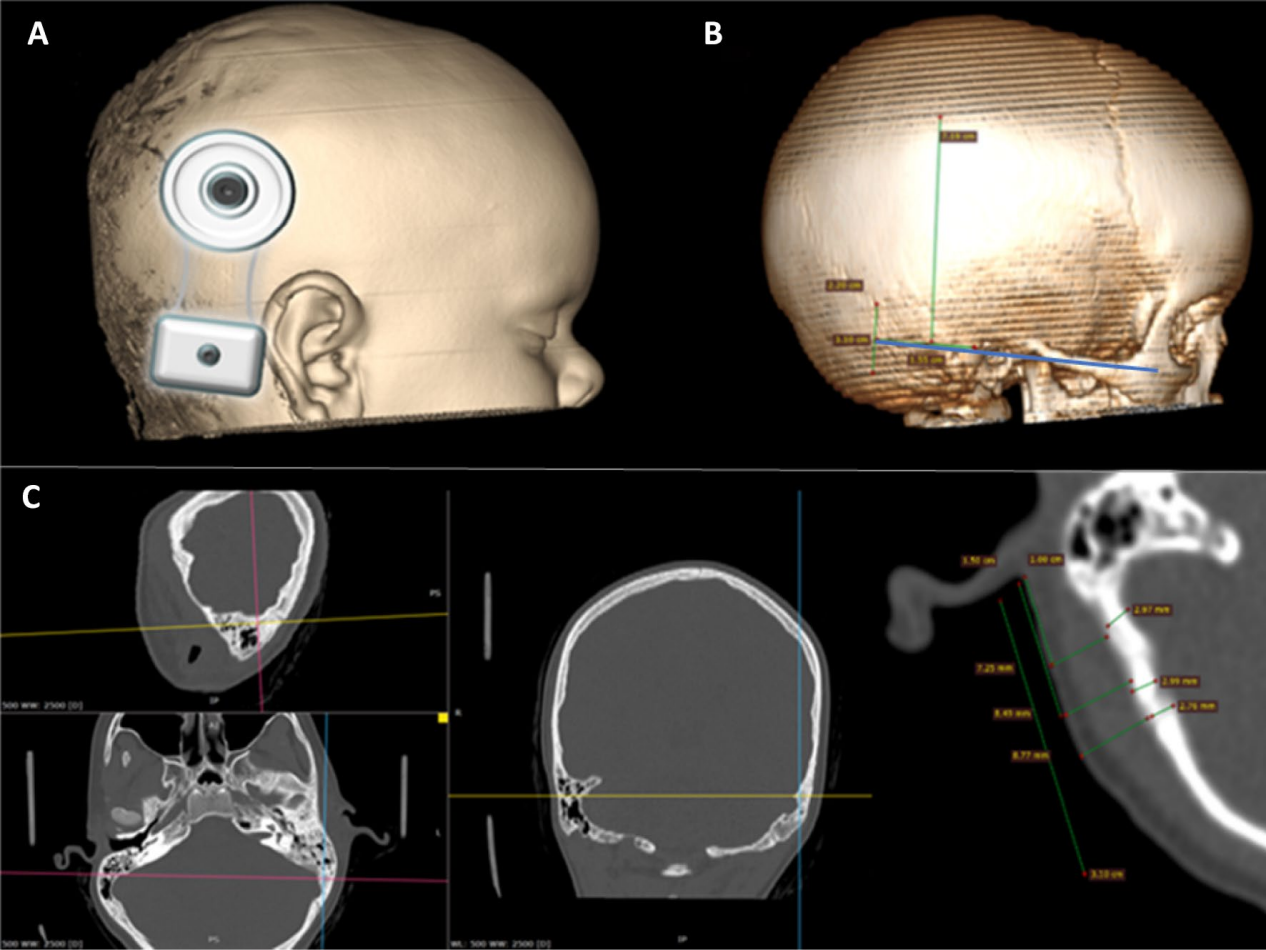
3D CT reconstruction of a 5-year-old male. Picture **A**: schematic position of the implant Picture **B**: reference lines determined by fix points: lower margin of the orbita, zygomatic arch and the ear canal midline (blue line). Green reference line shows the mid-axis of the implant. Picture **C**: multiplane view of cranial CT scans: fix points were set in each view. Soft tissue and bone thickness were calculated in the region of interest

All output of CT data were converted into Digital Imaging and Communications in Medicine (DICOM) files, and exported to RadiAnt DICOM viewer 2020.2 (Medixant, Poznań, Poland) for morphometrical studies. Based on the recommended position and dimensions of the implant (Fig. 1) soft tissue and bone thickness was determined. To achieve a reproducible measuring method, fix points were assigned: lower margin of the orbita, zygomatic arch and the external ear canal (EEC) midline. Implant parameters were marked on the reference lines defined by the fix points. Bone and soft-tissue thicknesses were calculated in a multi-plane view along the mentioned reference lines (Fig. 2) Three adjacent sample points were taken and average skin and bone thickness was determined in each session. With this method, soft-tissue thickness was assigned in the level of the SP and in the level of the transducer, while bone thickness was calculated in the level of transducer/possible position of BI300 implant, i.e., EEC midline. Besides the recommended BI300 position, bone thickness was also measured superior to the EEC midline to collect information of bone structure and to determine whether alternative placement of the titanium implant would be feasible. For safety purposes, sigmoid sinus distance from the posterior wall of the EEC, and bone thickness above the sinus was also observed. Both the left and right temporal areas were analysed of each patient. For further planning, representative samples were printed in 3D to verify our measuring technique.

Data were collected and statistically analysed with SYSTAT 13 Software (Inpixon Inc., Version 13. Palo Alto, United States). Results are presented as mean ± SD.

## Results

### Soft-tissue thickness

Table 1 shows the soft-tissue thickness at the level of the SP and transducer. Average soft-tissue thickness in the level of the SP in the entire population was 3.7 ± 0.6 mm, which was significantly lower compared to the level of the transducer 6.3 ± 2.2 mm (ANOVA, Mann–Whitney Rank Sum Test, *p* < 0.001). No difference between the left and right side was found. In the different age groups, no significant difference was found in the level of SP, however, in the level of transducer, soft-tissue thickness slightly reduced with age.

**Table 1:** Soft-tissue thickness at sound processor level and transducer level in different age groups in the area of interest

Soft-tissue thickness	Age (year)	Mean ± SD (mm)
Sound processor level	**Together**	**3.7 ± 0.6**
	5–6	3.2 ± 0.5
	7–8	3.2 ± 0.1
	9–10	3.6 ± 0.5
	11–12	3.6 ± 0.6
Transducer level	**Together**	**6.3 ± 2.2**
	5–6	7.3 ± 2.4
	7–8	6.8 ± 3.1
	9–10	5.9 ± 1.6
	11–12	5.6 ± 1.7

### Bone thickness

**Fig. 3:**
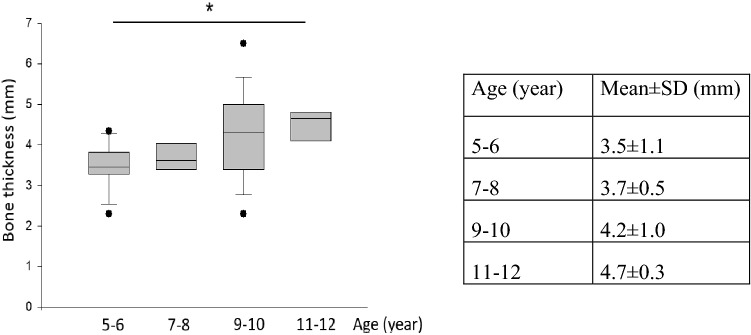
Average bone thickness in the recommended position of the implant in different age groups. (ANOVA, Mann–Whitney Rank Sum Test, *p* < 0.001)

Average bone thickness was 4.8 ± 1.6 mm in the recommended position of the BI300 implant (EEC midline) and 4.5 ± 1.2 mm at the level of the tegmen. However, at this level compact cortical bone was found in each age group, in contrast to the recommended position, where underlying mastoid cavity was found in 57% of cases. Significant differences were found in bone thickness between the youngest and eldest age groups, where average bone thickness was 3.5 ± 1.1 mm in those 5–6 years and 4.7 ± 0.3 mm in 11–12 years (ANOVA, Mann–Whitney Rank Sum Test, *p* < 0.001) in the recommended position (Fig. 3).

**Table:** 

Age (year)	Mean ± SD (mm)
5–6	3.5 ± 1.1
7–8	3.7 ± 0.5
9–10	4.2 ± 1.0
11–12	4.7 ± 0.3

### Sigmoid sinus

**Fig. 4:**
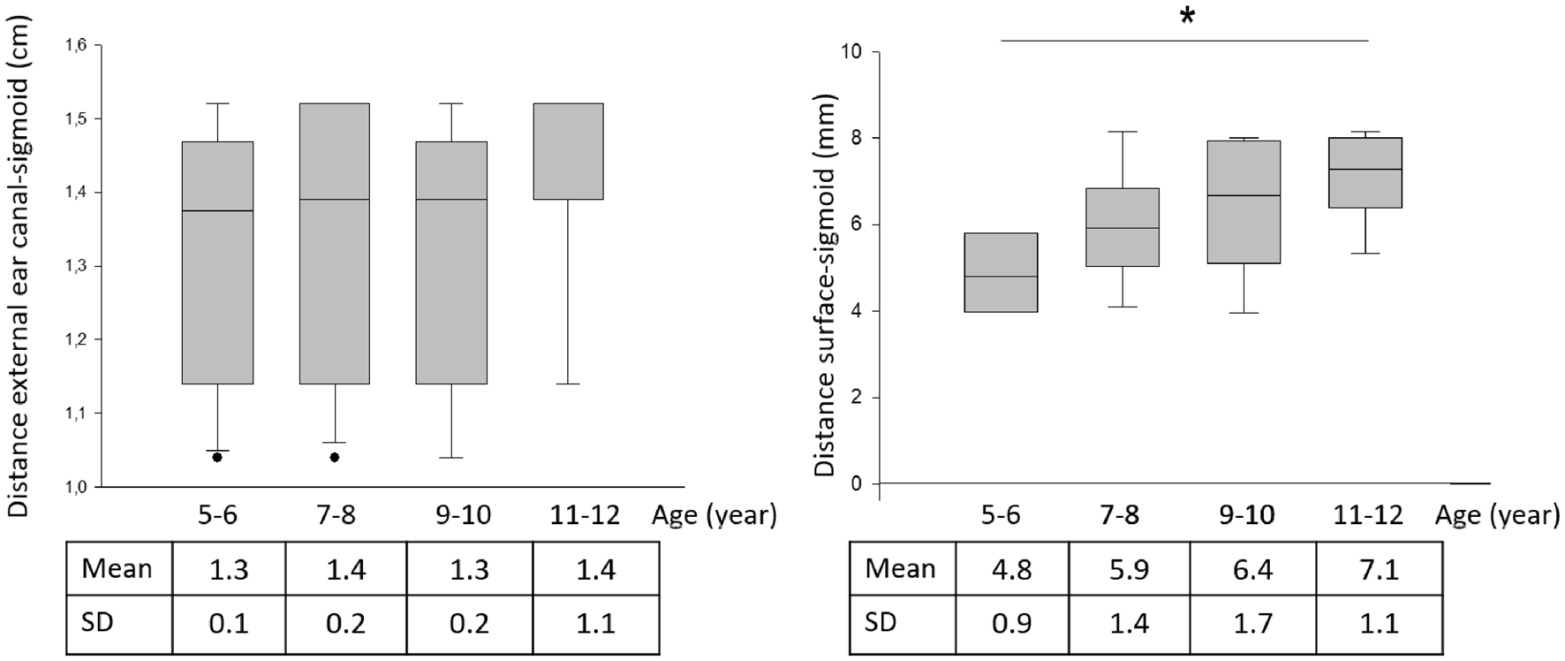
Linear dimensions of the mastoid by age group. Distance between the posterior wall of the external ear canal (left) and the anterior wall of the sigmoid sinus (right). No significant difference was found between the groups. In contrast, the distance between the bone surface and the upper wall of the sigmoid sinus increased with the age (*p* = 0.006). ANOVA, Mann–Whitney Rank Sum Test, *p* < 0.001)

Average distance of the anterior wall of the sigmoid sinus and posterior wall of the EEC was 1.3 ± 0.2 cm and no significant difference was found among age groups. In contrast, the distance between the bone surface and the bony sigmoid sinus wall increased with age and this was statistically significant (*p* = 0.006) (Fig. 4). However, these data suggest not only compact cortical bone, but perisinusoidal cells spreading above the sinus in 55% of cases (20% in 5–6 years, 40% in 7–8 years, 70% in 9–10 years and 90% in 11–12 years).

## Discussion

Early implantation of a hearing intervention in childhood can support effective hearing rehabilitation; however, surgery can be difficult due to the altered anatomy and dimension of the juvenile skull [14]. Preoperative CT for surgical planning, especially in young children, can be challenging and anaesthesia may be necessary. The aim of our study was to analyse several cranial CT scans of children between 5 and 12 years of age, to map the retroauricular area in different age groups and to help planning Osia implantation.

Implantation of Baha, more specifically implantation of BI300 does not essentially require preoperative CT in most of the cases, and the overall number of cranial CT scans in early childhood is small. Moreover, studies, help to predict the ideal implant size in children and determine the position of BI300, are rare. Although, especially in childhood, most common indication of BCIs are external ear canal/middle ear malformation and chronic otitis media; patients from non-otological cases were also selected to create an average population, similarly to other researcher groups [15, 16]. The idea behind our patient selection (i.e., otological and non-otological cases together) was, that previous study of pediatric uni- and bilateral ear canal atresia patients indicated that neither age nor diagnosis of atresia reliably predict a lower chance of identifying adequate bone thickness at typical implant sites, and no significant difference in bone thickness was found on the affected site compared to non-affected side [17]. Moreover, SSD patients do not necessarily have any anatomical involvement. In addition, hidden anatomical variations, different degree of mastoid air cell opacification, variation in mastoid pneumatisation resembles ventilation disorder also occurred in patient with no previous history of known ear problem, as we also perceived during the analysis.

To ensure a stable link between SP and coil in the Osia system, soft-tissue thickness should be under 9 mm at the level of the SP, Similar to Attract, 3–6 mm soft-tissue thickness is ideal [18]. In the Osia system the vibration is generated in the implanted transducer, so magnet force, vibration and consequent heating does not occur; therefore, the skin complications caused by strong magnet compression are reduced [19–21]. Since soft-tissue thickness was significantly below 9 mm in each age group, soft-tissue reduction could be avoided in children. In case of Attract, 3 mm flap thickness may adversely affect the risk of soft-tissue complication due to pressure, vibration and heat [22]. With the Osia system, the arrangement of the transducer and coil has solved this problem. In contrast with the passive Baha Attract, magnet strength in the Osia system is only necessary to hold the SP in place. At the transducer level, thicker tissue can reduce sound transmission with passive devices and lead to increased loads placed on the transducer to compensate for losses. Reduced tissue thickness in the older group may be due to the increasing size of the whole temporal area [23, 24].

It is also known that the size and shape of the mastoid develops continuously with age [23, 24]. However, most studies focus on the volume and shape of the mastoid cavity, which is important when large portions of the implant or the transducer have to be recessed. In a previous study of Rahne et al., many child mastoids were analysed to predict the probability of fitting Bonebridge in different age groups and to find the most ideal transducer shape. Nowadays, implantation softwares are also accessible to help preoperative planning of more robust implants (i.e., Bonebridge). These 3D methods give full detail of temporal bone density and volumetry [16, 25, 26]. Schilde et al. also highlighted that interindividual variation of temporal bone shape underlines the necessity of radiological preoperative planning in these cases [27]. An indisputable advantage of Baha systems, and the new Osia system, is that implantation needs minimal bone work. The ideal position of the Osia system is determined by the size of the transducer, which needs space behind the pinna and limits the position of the magnet and coil, as well as the SP. To accommodate differing bone thickness, different size (3 and 4 mm) BI300 titanium implants can be chosen. In our study, bone thickness was determined at different levels of the retroauricular space. Based on our results, the 3 mm BI300 is safe even in young children around the age of 5; and a 4 mm implant can be used in children aged 11–12. The possibility of entering the mastoid cavity increases with age due to the development of the air cells [23, 24]. Osseointegration in these cases is questionable, however, as the Osia system is transcutaneous, the possibility of tangential shear force, which can displace the system, is low compared to the percutaneous Baha Connect. Alternatively, positioning of the BI300 closer to the tegmen, where bone is more likely compact is advised. This can also be a good solution in cases, where the mastoid has previously been operated on, or where the possibility of future mastoidectomy is high.

For safety purposes, knowing the position of sigmoid sinus is also important. At the level of the recommended EEC midline the distance between the posterior wall of EEC and the anterior bony wall of the sigmoid sinus was relatively constant. However, the space between the sigmoid wall and the bone surface significantly increased, mainly due to developing mastoid cells. It is important to note that all our measurements were performed on a healthy population, without any severe malformations. In the study of Granström et al., possible dura and sigmoid sinus contact was found with 3 and 4 mm Branemark type (Nobel Biocare) implants in 26 and 11% of all 129 insertion cases, respectively. However, the age group was between 1 and 15 years, and a large number of patients had severe craniofacial malformations, which may influence mastoid cell formation and bone thickness [22]. Average bone thickness measured in 26 cases was also lower than in our study (2.5 ± 0.8 mm); however, the mean age of our study population was higher. Considering these findings, a 3 mm implant is the safest option for use in children.

The limitations of our study is the small sample size due to limited number of pediatric cranial CT scans; therefore, creation of much younger age groups or subgroups with different abnormalities within the age groups is challenging.

## Conclusion

Our study provides a basis for guiding Osia system implantation in the pediatric population. Based on our results, 3 mm BI300 implants are likely to be the best choice in pediatric cases, and a slight superior positioning of the implant may prevent breaching the mastoid air cells. Considering these findings, preoperative CT is unnecessary for Osia implantation in non-complicated cases. However, surgery of patients with complex craniofacial malformation might need more precise preoperative planning with CT imaging.
